# Update on the Role of the Endothelial Glycocalyx in Angiogenesis and Vascular Inflammation

**DOI:** 10.3389/fcell.2021.734276

**Published:** 2021-08-31

**Authors:** Zhengping Hu, Issahy Cano, Patricia A. D’Amore

**Affiliations:** ^1^Schepens Eye Research Institute of Massachusetts Eye and Ear, Boston, MA, United States; ^2^Department of Ophthalmology, Harvard Medical School, Boston, MA, United States; ^3^Department of Pathology, Harvard Medical School, Boston, MA, United States

**Keywords:** barrier, leukocytes, COVID-19, diabetes, endomucin

## Abstract

The endothelial glycocalyx is a negatively charged, carbohydrate-rich structure that arises from the luminal surface of the vascular endothelium and is comprised of proteoglycans, glycoproteins, and glycolipids. The glycocalyx, which sits at the interface between the endothelium and the blood, is involved in a wide array of physiological and pathophysiological processes, including as a mechanotransducer and as a regulator of inflammation. Most recently, components of the glycocalyx have been shown to play a key role in controlling angiogenesis. In this review, we briefly summarize the structure and function of the endothelial glycocalyx. We focus on its role and functions in vascular inflammation and angiogenesis and discuss the important unanswered questions in this field.

## Introduction and Function of the Endothelial Glycocalyx

### Structure

The apical surface of the endothelium, which lines the entire vasculature, is covered by a non-uniform, but complex structure comprised of glycosaminoglycans (GAGs) (primarily heparan sulfate, chondroitin sulfate, and hyaluronan) glycoproteins, glycolipids, and proteoglycans (predominantly syndecans and glypican), along with adsorbed proteins [such as albumin, antithrombin III, superoxide dismutase, and a number of growth factors, e.g., vascular endothelial growth factor (VEGFs) and fibroblast growth factors (FGFs)]. This structure is collectively referred to as the glycocalyx (sugar coat”: glykys = sweet, kalyx = husk). The thickness and composition of the negatively charged glycocalyx varies throughout the vasculature and is estimated to be between 0.5 and 5 μm thick, depending on methods used to measure the glycocalyx and at what level the vasculature it is being examined (Nieuwdorp et al., 2008). Interestingly, the glycocalyx has also been referred to as a “specialized extracellular matrix” (Moore et al., 2021), though one tends to think of an extracellular matrix as being on the basal side of a polarized cell layer such as the endothelium. In the broadest sense, one of the primary functions of the endothelial glycocalyx is to reduce access of circulating cells and plasma constituents to the vasculature (reviewed in Curry and Adamson, 2012).

As was once the case for the endothelium itself, the glycocalyx was initially considered to be a passive structure. However, an increasing body of research has revealed important roles for the glycocalyx in a wide range of homeostatic processes, reviewed below. The role of glycocalyx components in various disease processes and the effect of pathologies on the glycocalyx is discussed in section “Modulation of Glycocalyx Under Inflammation and Other Pathological Conditions.”

### Physiologic Functions

#### Anti-inflammation

Net negatively charged cells such as white and red blood cells and platelets are repelled from the glycocalyx due to its negative charge. Interactions between leukocytes and endothelial cell (EC) are mediated by adhesion molecules that direct leukocyte rolling along and adhesion to the endothelial surface. Under resting (non-activated) conditions these selectins and integrins are expressed at low or non-detectable levels and are masked by the overlying glycocalyx (Reitsma et al., 2007). The specific glycocalyx constituents responsible for preventing leukocyte adhesion have not been fully elucidated. At the level of the mouse intestinal mesentery, heparinase treatment results in increased leukocyte adhesion, pointing to a role for Heparan sulfate proteoglycans (HSPGs) (Mulivor and Lipowsky, 2002). In addition, using human umbilical vein ECs in culture, our group has shown that the knockdown of endomucin (EMCN) using siRNA leads to increased leukocyte adhesion in non-activated EC and that the adhesion was mediated by constitutively expressed I-CAM (Zahr et al., 2016). In addition, using the same system, the overexpression of EMCN in tumor necrosis factor-alpha (TNF-α) (activated) EC was able to significantly suppress leukocyte adherence as did adenoviral expression of EMCN in a mouse model of ocular inflammation.

#### Anti-thrombosis

A number of molecules bound to the glycocalyx account for its anti-coagulant and anti-thrombotic properties, including anti-thrombin III, tissue factor pathway inhibitor, and dermatan sulfate (reviewed in Potje et al., 2020). In addition, thrombomodulin, one of the glycocalyx proteoglycans, is another major contributor to this activity, acting via binding to thrombin (reviewed in Martin et al., 2013).

#### Permeability Control

Under normal conditions, the glycocalyx forms a barrier against vascular permeability, partly by acting as a negatively charged molecular sieve (Alphonsus and Rodseth, 2014). The glycocalyx impacts vascular permeability via its three-dimensional structure—interactions among the various components generate a “mesh-like” structure that can control passage of molecules based on their size. Its tight mesh structure excludes macromolecules greater than 70 kDa (Pries et al., 2000). In addition, the glycocalyx structure is negatively charged and thus can limit the transit of molecules based on charge. For instance, the glycocalyx is the primary site for the restriction of the passage of albumin across the endothelium (Curry and Adamson, 2012), which although net negatively charged binds to the glycocalyx due its amphoteric characteristics (reviewed in Aldecoa et al., 2020).

#### Mechanosensor

Heparan sulfate has been demonstrated to be a primary sensor of the directionality of shear stress. Exposure of cultured EC to heparinase increased the speed of flow-induced endothelial migration but prevented shear-stress stimulated directionality of migration and recruitment of phosphorylated focal adhesion kinase (Moon et al., 2005). The glycocalyx also transmits the effects of shear stress on endothelial nitric oxide synthase (eNOS), which is localized to caveolae on the endothelial surface. Enzymatic removal of heparan sulfate prevents sheer stress-induced NO production (Pahakis et al., 2007). Recent evidence indicates that it is heparan sulfate in the form of glypican-1 that mediates this signal, as depletion of glypican-1 using siRNA prevent the shear stress activation of eNOS (Ebong et al., 2014). Using syndecan-4 core protein as a model, a large-scale molecular dynamics computational experiment indicated the syndecan-4 core protein manifests as a scissors-like motion and transmits force as a main pathway of signal transmission. While blood flow velocity changes the shedding of proteoglycan sugar chain, its functional role is to protect the core protein from severe conformational changes (Jiang et al., 2020). More mechanistic roles of glycocalyx in mechanosensing and transduction have been thoroughly reviewed recently (Zeng et al., 2018).

#### Membrane Shape Regulation

A number of the proteoglycan core proteins span the plasma membrane and associate with the endothelial cortical cytoskeleton via linker proteins such as talin and dystonin, thus communicating extracellular forces though the cell (reviewed in Cosgun et al., 2020). Conversely, the cortical cytoskeleton can alter the conformation of the glycocalyx so that polymerized actin leads to a flattened glycocalyx and actin disassembly results in a more extended glycocalyx. A recent study has demonstrated cooperation between intracellular cytoskeletal components and constituents of the glycocalyx in how cells alter their shape in response to their microenvironment. Specifically, it was shown that “polymers” formed by glycocalyx mucins and polysaccharides “generate an entropic bending force to favor formation of curved membrane structures” (Shurer et al., 2019). The authors speculate that the morphological impact of the glycocalyx would have “broad consequences on membrane processes, ranging from absorption and secretion to cellular communication, signaling, and motility.”

#### Regulation of Growth Factor, Cytokine, and Chemokine Signaling

With heparan sulfate comprising the largest proportion of the glycocalyx (Reitsma et al., 2007) and knowing the significant effect that heparan sulfate has on the bioavailability, activity, and stability of a wide number of growth factors (Ricard-Blum and Lisacek, 2017), it is not surprising that the glycocalyx has an essential role in vessel formation and maturation. For example, heparan sulfate containing GAGs have been shown to interact with both the angiopoietins and Tie receptors as well as serving as a ligand for Tie1, enhancing signaling and cell survival (Griffin et al., 2021). HSPGs interact with VEGF and promotes angiogenesis through increasing VEGF levels in the extracellular microenvironment and fine-tuning the interaction of VEGF with its receptor and co-receptor (Nagarajan et al., 2018). Another membrane glycoprotein, neuropilin, is known to function as a co-receptor for VEGF receptor 2 (VEGFR2) and guide developmental angiogenesis (Gelfand et al., 2014). Using zebrafish as a model, it was demonstrated that fish lacking perlecan failed to develop primary intersegmental vessel sprouts (Zoeller et al., 2008). In addition, work from our group has identified a role for EMCN, a type 1 transmembrane glycoprotein abundantly expressed by venous and capillary endothelium, in the regulation of VEGFR2 signaling (see Part 2 below on glycocalyx and angiogenesis). Chemokines-GAG interactions mediates chemokines membrane localization and surface presentation and fine-tune the function of chemokines (Proudfoot et al., 2017). Interaction between CXCL8 and extracellular HSPGs regulates the leukocyte recruitment and transmigration (Sarris et al., 2012).

#### Regulation of the Glycocalyx

One major regulator of glycocalyx thickness is flow, such that exposure of culture EC to laminar flow leads to the increased synthesis of hyaluronan (Maroski et al., 2011). Whereas disturbed flow induces an approximately 50% decrease in endothelial glycocalyx compared to uniform laminar flow *in vitro* (Mensah et al., 2020). This effect of shear stress on the glycocalyx becomes particularly relevant in the pathogenesis of atherosclerosis (see Part 3). Exposure to pro-inflammatory cytokines leads to a dramatic reduction in the glycocalyx, largely by endothelial-derived metalloproteinase-mediated shedding (Yang et al., 2020) as well as by heparinase secreted by mast cells and hyaluronidase produced by the EC (Bourguignon and Flamion, 2016).

## The Glycocalyx in Angiogenesis

### Role of Glycocalyx in Angiogenesis

#### Normal Angiogenesis

Angiogenesis is the formation of blood vessels from pre-existing vessels, and components of the glycocalyx mediate elements of angiogenesis in diverse and multifaceted ways. GAGs play an active role in regulating angiogenesis. In general, the gel-like composition generated by the presence GAGs, in combination with identified cytokine interactions, mainly FGFs and VEGF, facilitate growth factor signaling in ECs by influencing their bioavailability, local concentrations, and stability (Muramatsu and Muramatsu, 2008; Huynh et al., 2012).

Proteoglycans have been implicated in modulating angiogenesis more specifically by influencing cell proliferation and migration under both normal and pathologic conditions (Iozzo and Sanderson, 2011; Gubbiotti et al., 2020). Heparan sulfate is a known regulator of angiogenesis, and its 6-O-sulfation specifically has been reported to modulate VEGF165-induced angiogenesis (Stringer, 2006). VEGF, a well-studied, angiogenic factor, is produced as a number of splice variations, which differ in their ability to bind to heparan sulfate proteoglycan. Whereas VEGF120 does not contain any heparan sulfate binding domains, it is freely soluble upon its secretions from cells, VEGF188, on the other hand, binds very tightly to heparan sulfate and remains cell-associated upon release, and VEGF164 displays intermediate properties. Mice genetically engineered to express only VEGF120, that is lacking both heparan sulfate binding isoforms, are lethal perinatally due to defective lung development (Galambos et al., 2002), indicating the importance of growth factor association with the glycocalyx to their function.

Members of syndecan family of heparan sulfate proteoglycans have been studied for their diverse roles in angiogenesis regulation. Various syndecans have distinct function in promoting or inhibiting angiogenesis depending on whether they are present on the EC extracellular membrane or shed (De Rossi and Whiteford, 2013; De Rossi et al., 2014). Syndecan-1 knockdown in both *in vivo* and *in vitro* has been shown to interrupt VEGFR2 internalization by preventing clathrin-mediated endocytosis (Jing et al., 2016). A decrease in VEGFR2 expression following syndecan-1 depletion has also been reported (Lamorte et al., 2012). Syndecan-2 is necessary for angiogenesis, potentially acting as a co-receptor for VEGF signaling, giving it a critical role in promoting angiogenesis whereas the shed variant of the protein plays an active role in inhibiting cell migration (Essner et al., 2006; De Rossi et al., 2014). Similarly, syndecan-4 binds VEGF “sequestering” and preventing it from exerting its angiogenic signaling (Lambert et al., 2020).

Hyaluronan (HA), an abundant glycocalyx GAG, exerts an anti-angiogenic effect in its native form, whereas its degradation products induce angiogenesis by activating HA receptors CD44 and CD168 (West and Kumar, 1989; Slevin et al., 2007; Ghose et al., 2018). HA has also been reported to prevent the binding of VEGF to its primary signaling receptor VEGFR2 in a sulfate-dependent manner (Rother et al., 2017).

Another prominent glycocalyx component is EMCN, a membrane spanning mucin-type glycoprotein that has been shown to be involved for VEGFR2 signaling. Initial studies demonstrated that the knockdown of EMCN in the developing vasculature led to impaired vascular development and reduced tip cell activity (Park-Windhol et al., 2017). Investigation of the mechanism underlying this effect using primary human retinal capillary EC in culture revealed that siEMCN knockdown of EMCN blocked VEGF-stimulated proliferation, migration, and tube formation. Subsequent studies showed that the lack of EMCN leads to a failure of VEGF-activated VEGFR2 to internalize, thereby preventing the sustained intracellular signaling that is necessary for VEGF to stimulate endothelial function. EMCN, however, is itself not internalized with VEGFR2 during normal signaling (LeBlanc et al., 2019). Structure-function analysis using EMCN truncation mutants has revealed the minimum extracellular 21–121 amino acids of EMCN necessary for modulating VEGFR2 signaling and indicated also that the activity is dependent on N-glycosylation (Hu et al., 2020).

#### Pathologic Angiogenesis

Since the first description of the animal glycocalyx by Martínez-Palomo (1970), there has been growing interest in the role of the glycocalyx, including in pathologic angiogenesis. Recent reports demonstrate that the glycocalyx is important for not only developmental angiogenesis and its maintenance, but for cancer progression secondary to its vascularization as well. Angiogenesis is necessary for tumor growth beyond the limits of diffusion. The modification of the glycocalyx structure of cancer cells has been suggested to provide tumors with the ability to survive and overcome vascular limitations, for example by mimicking the vasculogenic nature of ECs with 3-dimentional organization, while lacking EC markers such as CD31 and CD34 (Tachi et al., 2019). FGF and VEGF contain heparan-binding domains and their signal transduction depends on its sulfation. Alteration of heparan sulfate’s ability to bind FGF and VEGF has been reported to reduce angiogenic signal transduction and weaken the vascular foundation for tumors (Fuster et al., 2007). As the specific components of the glycocalyx are actively involved in aberrant angiogenesis, they may represent promising targets for therapy.

The stability of the vasculature is also dependent on the integrity of the glycocalyx layer as seen in diabetic retinopathy. Hyperglycemia has been reported induce the shedding of various components of the glycocalyx, disrupting EC function by increasing permeability in glomerular ECs (Singh et al., 2011). Loss of EMCN, for example, has been suggested to be associated with diabetic retinopathy and the overexpression of EMCN leads to the restoration of the glycocalyx and the related EC function in streptozotocin-induced diabetic rats (Niu et al., 2019). VEGF165b, a non-angiogenic isoform of VEGFA, was reported to restore the glycocalyx and ultimately the endothelium after diabetic damage in isolated diabetic human and rat glomeruli. VEGF165b shares the protective prosurvival and antiapoptotic signaling of canonical VEGF isoforms and was reported to restore the glycocalyx in short and long-term conditions (Oltean et al., 2015).

## Modulation of Glycocalyx Under Inflammation and Other Pathological Conditions

**FIGURE 1:**
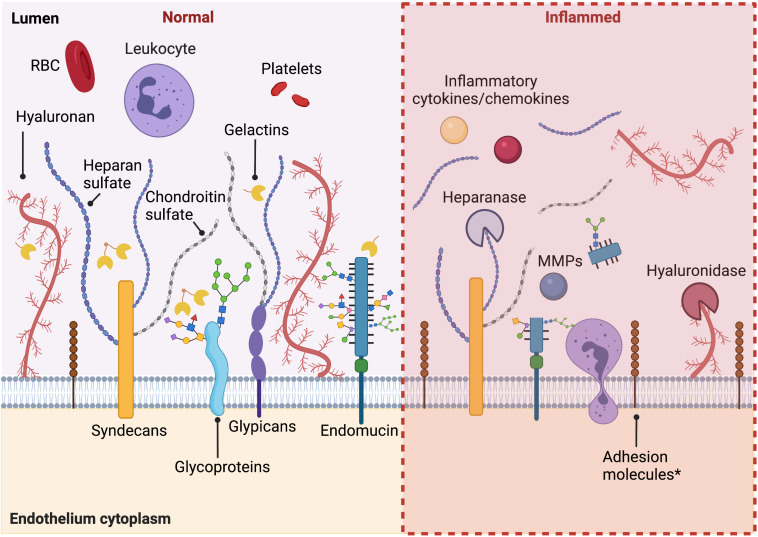
Summary of the modulation of glycocalyx under activated conditions. Under healthy conditions, intact endothelial glycocalyx maintains endothelium homeostasis, regulating permeability, and plays an anti-inflammation and anti-thrombosis role. When activated in pathological conditions, during inflammation, diabetes, and other pathological conditions, there is increased shedding of the glycocalyx. An increased expression of adhesion molecules under inflammatory conditions is also observed. Figure was created with BioRender.com.

As described above, the glycocalyx protects endothelial cells by acting as a barrier between circulating blood cells and the endothelium, regulating permeability, controlling nitric oxide production, and functioning as a mechanosensor. Systemic and local inflammatory responses, including diabetes, atherosclerosis, surgical ischemia/reperfusion injury, and sepsis, have been shown to cause rapid loss of glycocalyx and its activities, both directly and indirectly. A summary of the modulation of glycocalyx under activated conditions is shown in Figure 1.

### Inflammation

The vascular endothelium is the earliest site of involvement in the systemic inflammatory response syndrome (Becker et al., 2010). Proinflammatory stimuli lead to dramatic alterations in the thickness of the glycocalyx. For instance, during sepsis the thickness of the glycocalyx decreases by up to 66% *in vivo*, measured by intravital microscopy (Kennett and Davies, 2007). Shedding of the glycocalyx during inflammation has been reported in many studies and its breakdown is accompanied by shedding of one or more glycocalyx components into circulation including syndecan-1, heparan sulfate, and HA (Dogne and Flamion, 2020). Moreover, markers of endothelial glycocalyx shedding, like heparan sulfate and syndecan-1, have been found increased in both plasma and urine in sepsis patients (Schmidt et al., 2016), and shown to correlate with disease severity (Anand et al., 2016). Increased plasma levels of endothelial surface layer components in septic patients are positively correlated with increased mortality (Becker et al., 2010).

The damage to the endothelial glycocalyx noted during inflammation is initiated by the actions of TNF-α, bacterial lipopolysaccharides and other cytokines on the endothelium, which leads to the production and secretion of endothelial-derived metalloproteinases and heparinases that mediate the shedding of glycocalyx (Tarbell and Cancel, 2016). In addition, mast cells are activated by TNF-α and release cytokines, proteases, histamine, and heparinase, further degrading the glycocalyx. Concomitant with the cytokine-induced degradation of the glycocalyx exposing constitutively expressed adhesion molecules including I-CAM, the cytokines also stimulate the expression of selectins and integrins to facilitate rolling and adhesion of monocytes and polynuclear neutrophils followed by diapedesis (Nelson et al., 2008). With the altered glycocalyx no longer able to perform its normal functions, there is increased vascular permeability, tissue edema, increased leukocyte adhesion, platelet aggregation, and dysregulated vasodilation (Becker et al., 2015).

### Diabetes

Diabetes is another major pathology in which the degradation of the glycocalyx has been implicated, as demonstrated in type 1 and type 2 diabetics in both animal models as well as in humans (Broekhuizen et al., 2010; Leskova et al., 2019). Experimental evidence indicates the involvement of reactive oxygen species, advanced glycation end products (AGEs), and activation of the sheddases heparanase and hyaluronidase in glycocalyx deterioration during diabetes (Dogne et al., 2018). Overproduction of superoxide anion results in increased oxidative stress, which leads to a depolymerization of GAGs, particularly HA, as well as AGE formation (Kennett and Davies, 2009). AGE levels were significantly higher in vitreous and serum of patients with diabetic retinopathy compared to normal controls (Sebag et al., 1994) and AGEs have been shown to promote the degradation of HA *in vivo* (Katsumura et al., 2004). Heparinase, secreted by macrophages and activated podocytes, is also well documented to be involved in the process of endothelial glycocalyx degradation and diabetic nephropathy (Boels et al., 2016), and, matrix metalloproteinases (MMPs), including MMP-2 and MMP-9, have been implicated in glycocalyx changes seen during the progression of diabetic retinopathy (Eshaq et al., 2017). In immortalized human glomerular EC, IL-1β and TNF-α increased MMP-9 mediated shedding of cell surface heparan sulfate and syndecan-4, measured by western blot (Ramnath et al., 2014). In addition, there are increased levels of plasma hyaluronidase, as measured by *ex vivo* enzymatic activity, in the streptozotocin-induced mice diabetes model. Deficiency of hyaluronidase in knockout mice resulted in a thicker glycocalyx and protection from HA shedding (Dogne et al., 2016).

### COVID-19

Endothelial glycocalyx damage has been recently reported in COVID-19 patients (Buijsers et al., 2020; Fraser et al., 2020; Stahl et al., 2020; Rovas et al., 2021). A number of the features of COVID-19 that are not been seen in other viral infections including, asymptomatic pneumonia, acute respiratory distress syndrome (Kazory et al., 2020), disseminated intravascular coagulation (Guan et al., 2020), rapid progression, and sudden death associated with thromboembolism, all appear to be by-products of known glycocalyx functions. Thus, it has been suggested that COVID-19 is an endothelial disease, particularly in its later stages, which often involve a “cytokine storm” that shifts endothelial functions into a “defensive mode” (Libby and Luscher, 2020). In light of the role of the endothelial glycocalyx in vascular homeostasis, including vascular permeability and vessel tone as well as an anti-thrombotic and anti-inflammatory (Yang et al., 2020), it appears that levels of glycocalyx components may be employed as a biomarker for the severity of COVID-19 and as an indicator of therapeutic response. Patients on mechanical ventilation had severe damage to the glycocalyx, with the glycocalyx being thinner as measured by perfusion boundary region and higher blood levels of shed glycocalyx constituents such as hyaluronic acid (Ding et al., 2020), chondroitin sulfate (Fraser et al., 2020), and syndecan-1, a transmembrane heparan sulfate proteoglycan (Rovas et al., 2021). Levels of heparanase, known to degrade the endothelial glycocalyx, were found to be considerably higher in COVID-19 patients than healthy controls and the levels of heparanase activity are linked to the severity of COVID-19 disease (Buijsers et al., 2020).

## Unanswered Question and Challenges

Our understanding of the structure and function of the glycocalyx has evolved tremendously over the last few decades. One challenge is obtaining proper visualization of the glycocalyx. Early imaging of the structure led to the underestimation of the thickness of the glycocalyx layer (Reitsma et al., 2007; Dane et al., 2015). The glycocalyx structure is too fragile for common sample preparation techniques, and degradation and dehydration of the layer is common. Alternatives methods for preparation, such as rapid freezing, have provided more accurate dimensions for the glycocalyx, 100-fold thicker in some regions than previously reported (Ebong et al., 2011).

Despite increasing evidence of the biological relevance of endothelial glycocalyx in various pathological conditions, detailed mechanisms of its role and regulation are difficult to dissect at the molecular level. Although new scientific techniques have allowed for a better understanding and detection of endothelial glycocalyx derangement and more components of glycocalyx are being used as markers in pathological conditions, the intrinsic complexity of the glycosylation process and the enormous diversity of glycan structures combined with the technical limitations of the current experimental tools for modulating the structure and expression of glycoproteins are among the challenges faced in the study of the endothelial glycocalyx (de Haas et al., 2020).

## Author Contributions

ZH, IC, and PD’A shared equally in writing the manuscript. All authors contributed to the article and approved the submitted version.

## Conflict of Interest

The authors declare that the research was conducted in the absence of any commercial or financial relationships that could be construed as a potential conflict of interest.

## Publisher’s Note

All claims expressed in this article are solely those of the authors and do not necessarily represent those of their affiliated organizations, or those of the publisher, the editors and the reviewers. Any product that may be evaluated in this article, or claim that may be made by its manufacturer, is not guaranteed or endorsed by the publisher.
